# Clinical chemistry reference intervals of healthy adult populations in Gojjam Zones of Amhara National Regional State, Northwest Ethiopia

**DOI:** 10.1371/journal.pone.0184665

**Published:** 2017-09-08

**Authors:** Zewdie Mekonnen, Asmare Amuamuta, Wondemagegn Mulu, Mulat Yimer, Yohannes Zenebe, Yesuf Adem, Bayeh Abera, Wondemu Gebeyehu, Yakob Gebregziabher

**Affiliations:** 1 Department of Biomedical Research, Biotechnology Research Institute, Bahir Dar University, Bahir Dar, Ethiopia; 2 Department of Biochemistry, College of Medicine and Health Sciences, Bahir Dar University, Bahir Dar, Ethiopia; 3 Department of Microbiology, Parasitology and Immunology, College of Medicine and Health Sciences, Bahir Dar University, Bahir Dar, Ethiopia; 4 Amhara National Regional State Public Health Institute, Amhara National Regional State Health Bearue, Bahir Dar, Ethiopia; Oregon State University, UNITED STATES

## Abstract

**Background:**

Reference interval is crucial for disease screening, diagnosis, monitoring, progression and treatment efficacy. Due to lack of locally derived reference values for the parameters, clinicians use reference intervals derived from western population. But, studies conducted in different African countries have indicated differences between locally and western derived reference values. Different studies also indicated considerable variation in clinical chemistry reference intervals by several variables such as age, sex, geographical location, environment, lifestyle and genetic variation.

**Objective:**

This study aimed to determine the reference intervals of common clinical chemistry parameters of the community of Gojjam Zones, Northwest Ethiopia.

**Method:**

Population based cross-sectional study was conducted from November 2015 to December 2016 in healthy adult populations of Gojjam zone. Data such as, medical history, physical examination and socio-demographic data were collected. In addition, laboratory investigations were undertaken to screen the population. Clinical chemistry parameters were measured using Mindray BS 200 clinical chemistry autoanalyzer as per the manufacturer’s instructions. Descriptive statistics was used to calculate mean, median and 95^th^ percentiles. Independent sample T-test and one way ANOVA were used to see association between variables.

**Results:**

After careful screening of a total of 799 apparently healthy adults who were consented for this study, complete data from 446 (224 females and 222 males) were included for the analysis. The mean age of both the study participants was 28.8 years. Males had high (P<0.05) mean and 2.5^th^-97.5^th^ percentile ranges of ALT, AST, ALP, creatinine and direct bilirubin. The reference intervals of amylase, LDH, total protein and total bilirubin were not significantly different between the two sex groups (P>0.05). Mean, median, 95% percentile values of AST, ALP, amylase, LDH, creatinine, total protein, total bilirubin, and direct bilirubin across all age groups of participants were similar (P>0.05). But, there was a significant difference in the value of ALT (P<0.05). The reference intervals of ALT, total protein and creatinine were significantly (P<0.05) high in people having monthly income >1500 ETB compared to those with low monthly income. Significant (P<0.05) higher values of the ALT, ALP and total protein were observed in people living in high land compared to low land residences.

**Conclusion:**

The study showed that some of the common clinical chemistry parameters reference intervals of healthy adults in Gojjam zones were higher than the reference intervals generated from developed countries. Therefore, strict adherence to the reference values generated in developed countries could lead to inappropriate diagnosis and treatment of patients. There was also variation of reference interval values based on climate, gender, age, monthly income and geographical locations. Therefore, further study is required to establish reference intervals for Ethiopian population.

## Introduction

Health and disease can be distinguished by accurate and reliable reference intervals of a clinical laboratory testing [1]. Reference interval is crucial for disease screening, diagnosis, monitoring, progression and treatment efficacy. Clinical chemistry reference intervals are also important tool for identifying abnormal laboratory results and ultimately guiding patient management decisions [2]. Reference intervals are typically established by assaying specimens from a sample group of people who meet carefully defined criteria [3–6]. Reference interval is usually defined as the values encompassing the central 95% of specimens; equating to 2 standard deviations on either side of the mean [2,7]. Producing reference intervals for a general population is a major challenge, as it requires selecting the appropriate reference population and recruiting individuals who represent relevant demographic groups that meet the inclusion criteria; collecting, processing and testing specimens; and finally, calculating reference values with possible stratification of the data into subgroups [7].

Clinical chemistry parameters vary considerably in terms of age, sex, life style, environment and genetic factors [6]. Some studies conducted in Asian and African countries also showed differences in the reference values compared to the established western references as presented elsewhere and considerable differences in the reference values by sex among population groups exist [6]. Studies conducted in other countries also addressed that ethnic origin, genetics, gender, dietary patterns, altitude and environmental factors influence some values of biochemical indices, suggesting that the development of reference values for the African population may be beneficial for improved quality of health care [6].

The significant difference in the reference intervals of clinical chemistry parameters among different countries and population groups within the same country may increase the risk of either unnecessary additional investigations or failure to detect underlying disease or mismanagement of patients [5, 6, 8]. Due to lack of enough data at a population level in the Ethiopian situation, we are using reference values of clinical chemistry parameters generated from populations of developed countries. In addition, there is no enough data on the clinical chemistry parameters reference interval in Ethiopia and in Amhara National Regional State. Determining the reference intervals of common clinical chemistry tests in healthy individuals of the Amhara National Regional State is important to know the trends in clinical practice for the assessment of health and disease. Therefore, there is an urgent need to have a base line data of reference intervals for common clinical chemistry parameters for healthy population at the community level. Thus, this study was aimed to determine the reference intervals of common clinical chemistry parameters in the community of Gojjam Zones, Northwest Ethiopia.

## Materials and methods

### Study design and period

A community based cross-sectional study was conducted from November 2015 to December 2016 among healthy adult populations in Jabitehnan and Debremarkos administrative woredas (districts) of Gojjam zones, Amhara National Regional State, Northwest Ethiopia.

### Study area and population

**Fig 1:**
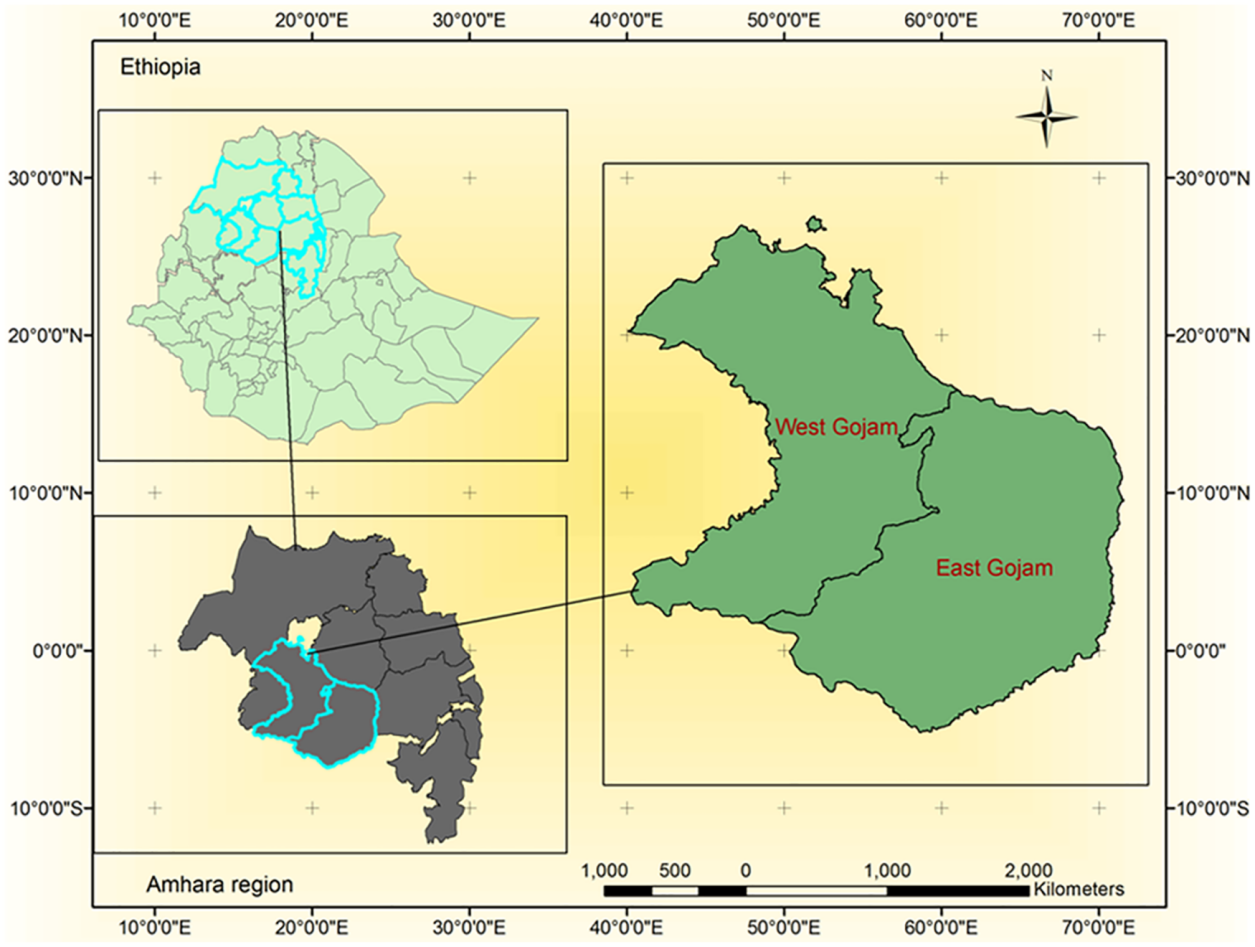
Map of the study population area.

The state of Amhara is located in the north western and north central part of Ethiopia (Fig 1). It has an area of 157,347sq.km [9]. The State shares common borders with the state of Tigray in the north, Afar in the East, Oromiya in the south, Benishangul/Gumuz in the south west, and the Republic of Sudan in the west. According to the 2007 census, the region's population was 17, 697, 272 of which males were a little bit higher than females [9].

Based on the 2007 census result, of the total population of the state, 81.5% were Orthodox Christians, 18.1% Muslims and 0.1% Protestants. In addition to the Amhara (which accounted around 91.2% of the population), Oromo (3%), Agew/Awi (2.7%), Kimant (1.2%), and Agew/Kamyr (1%). are included in the ethnic compositions of the region. The region is divided into 11 zonal administrations with 140 woredas (districts). There are about 3429 kebeles (neighbourhoods). Out of these, 118 are rural and 22 are town administration [9]. Jabitehnan woreda (district) is a lowland area located in West Gojjam Zone, 160 Kilometers away from the regional city. It has a total population of 287,045. Debremarkos is the capital city of East Gojjam zone located 268 Kilometers away from Bahir Dar city. It has an altitude of ≥ 1800 meter above sea level with a total population of 93,902 [9]. The source populations were all healthy adult populations of the East and West Gojjam Zones in the Amhara National Regional State. Healthy adults with age ≥ 18 years of both sexes from the selected house hold in the study area were the study population.

### Sample size and sampling techniques

The sample size was calculated using single proportion population formula [10]. Because of the lack of data on reference intervals of different clinical chemistry parameters at a regional as well as national level, a proportion of 0.5 mean (μ) population for each parameters, 2% marginal error, 95% confidence level and 15% non response rate were considered to calculate the sample size. Considering altitude difference, one study area from lowlands and another one area from the highlands were randomly selected from Gojjam administrative zone. The determined sample size was allocated for each selected areas proportional to their population size. From the two selected woredas (districts), the number of kebele (neighbourhood) was determined proportional to their population size. The determined kebeles (neighbourhoods) were selected using simple random sampling technique. Moreover, from the selected kebeles (neighbourhoods), the number of household was determined proportional to their population size. The households from each kebele (neighbourhood) were selected every n^th^ using systematic random sampling after getting the n^th^ value, by dividing the total number of households with the total number of selected households. From each selected households, adults (≥18 years old) who were healthy based on the inclusion criteria were selected using lottery method and one adult from each house hold was included in the study.

### Variables

Reference intervals of common clinical chemistry parameters were the dependent variable whereas sex, age, geographical location (altitude), life style, ethnicity, religion and residence were the independent variables.

#### Inclusion criteria

Healthy individuals (adults) with age ≥ 18 years of both sexes from the selected households of Gojjam zones of the Amhara National Regional State, Northwest Ethiopia.

#### Exclusion criteria

Age <18 years, adults with common intestinal parasitic infections, hemoparasite, skin rashes, history of blood transfusion < 6 months, HIV, HCV, HBV positives and HCG positive (for females), observable mental illness, disabled, smokers, chronic alcohol drunkers, anaemic, malnourished (BMI<17.5Kg/m^2^), hospitalized persons, chronic diseases and acutely ill as per the recommendations of WHO were excluded from the study.

### Operational definition

#### Healthy adults

Individuals (adults) with age ≥ 18 years and without disease or disabilities based on clinical sign and symptom plus laboratory investigations.

#### Reference intervals

The range between, and including two reference values defined by a specific percentage (usually 95%) for common clinical chemistry parameters of healthy individuals.

#### 95^th^ percentile ranges

It is the range between, and including the 2.5^th^ percentile and the 97.5^th^ percentile.

### Study procedure and data collection

The regional, zonal and woreda (district) health bureaus were communicated about the purpose of the study. Awareness creation was done for laboratory technologists, health officers, general practitioners, health extension workers, coordinators and other health care takers about the general study procedure to be followed. Moreover, together with the officers in the selected kebeles (neighbourhoods), health extension workers were sensitized for the study. In the regional health extension package program, the kebeles (neighbourhoods) in each woreda (district) were handled by health extension workers. The households were distributed to the health extension workers. The purpose of the study was communicated to the participants in the selected households and their willingness was confirmed. The informed study participants with no known chronic and acute disease and illness were transported to the nearby health centre.

In the health centre, these apparently healthy participants were screened by obtaining medical history and physical examination by physician. Those who passed this screening stage were further screened for intestinal parasitosis, diabetes, anaemia, HIV, HBsAg and anti-HCV. Moreover, pregnancy screening test (HCG test) was done using urine sample for all child bearing age women. Volunteers who screened positive for any of the transmissible infections, parasite, donated or received blood transfusion in the previous month and those who failed to give consent were excluded from the study. Results of HIV test were given to all participants after post-test counselling by trained HIV counsellor at the health centre. Quantitative data such as socio-demographic factors were collected from each participants using predesigned and structured questioner via face to face interview. After completion of the interview, all respondents were selected for clinical chemistry tests. About 5ml of venous blood was drawn from healthy adults in the morning using vacutainer system and transferred in to serum separator tube. Subsequently serum was separated in the separator tube. Then, test tubes were placed in ice-box and transported to the nearby hospital laboratory (Deberemarkos Referal Hospital) in the afternoon. Measurement of the clinical chemistry parameters was done within 8 hours of blood draw.

### Laboratory analysis

Common clinical chemistry parameters such as ALT, AST, ALP, amylase, LDH, creatinine, total protein, direct and total bilirubin values were done by using Mindray BS 200 clinical chemistry autoanalyzer (Germany) as per the manufacturer’s instructions.

### Quality control

To ensure the accuracy and precision of the test results, all pre-analytical, analytical and post-analytical precautions were taken into consideration. The Mindray BS 200 analyzer (Germany) and the protocols used were under regular control of the regional (ARPHI) and the national public health institute (EPHI). Moreover, all the laboratory staff received equipment and procedure (protocol) training from highly trained personnel working under the supervision of the regional (ARPHI) and the national public health institute (EPHI). The results obtained from the laboratory staff were validated and verified by trained personnel before release. In addition, to maintain internal quality control, known standards were run and the equipment was calibrated prior to analysis. The quality control results also included running two levels of controls (pathological/abnormal and non-pathological/normal) daily. The result of the two levels of controls had to be within acceptable ranges before testing samples. As external quality control, the laboratory also participates in the international digital Proficiency Testing (PT) program every three month.

### Data management and statistical analysis

All statistical calculations were performed on the Statistical Package for Social Sciences (SPSS) version 20.0 software (IBM Corp- Released 2011. IBM SPSS statistic Armonk, NY: IBM Corp). Descriptive statistics was used to determine the mean, median and 95% range of each parameters. Independent sample T-test and one-way ANOVA were employed to see the association between variables. All statistical tests were two tailed, and P-value < 0.05 considered statistical significant.

### Ethical clearance

Ethical clearance was obtained from the research ethics review committee of Biotechnology Research Institute of Bahir Dar University. Written consent was obtained from each study participants. All participants diagnosed for any illness were treated accordingly. Information obtained at any course of the study was kept confidential.

## Results

### Screening results

Table 1 described the characteristics of the study participants. A total of 799 apparently healthy adults consented for this study. After carefully screening, complete data from 446 (224 females and 222 males) were included for analysis. After screening, those who were HIV positive, HBV, HCV positive and positive for other parasitosis were referred to the standard care and not included in the study.

**Table 1:** Demographic characteristics of study participants.

Sex	Frequency (Number)	Percent
** Male**	222	49.8
** Female**	224	50.2
**Age (in Years)**		
** 18–24**	205	46.0
** 25–34**	115	25.8
** 35–44**	61	13.7
** 45–54**	32	7.17
** 55–64**	18	4.04
** ≥65**	15	3.36
**Educational status**		
** Illiterate and Elementary Completed**	183	41.0
** High School Completed and Above**	263	59.0
**Altitude**		
** High land**	172	38.6
** Low land**	274	64.4
**Income**		
** ≤ 1500ETB**	381	85.4
** ˃1500ETB**	65	14.6
**Marital status**		
** Single**	229	51.3
** Married**	217	48.7

### Demographic characteristics

The mean age of both the study participants at the study entry period was 28.8 years. The mean age of the females and males were 29.5 and 27.7 years respectively. Majority of (61.4%) the participants were from lowlands. In regards to educational level, 59% of the participants were high school complete and above. In terms of marital status, 229 (51.3%) were single and 217 (48.7%) were married.

### Clinical chemistry reference intervals

The calculated mean, median, 95% CI for mean and 2.5^th^-97.5^th^ percentile range values of clinical chemistry parameters based on sex, age, educational status, monthly income and climate were summarized in Tables 2, 3, 4, 5 and 6, respectively. The overall mean value of ALT, AST, ALP, amylase and LDH of participants were 18.0 U/L, 21.7U/L, 160.9 U/L, 139.9 U/L and 323.6 U/L respectively. Males had reference intervals of ALT 6.0–44.6 U/L against females of 3.0–30.0 U/L, AST value of 10.5–39.0 U/L against females of 6.0–32.1 U/L, ALP of 55.3 U/L-273.2 U/L against females of 49.0–236.0 U/L, amylase of 45.3–190.0U/L against females of 48.0–187.9U/L and LDH of 146.1–402.0 U/L against females of 137.7–405.1U/L. Males had significantly(p<0.05) higher 2.5^th^-97.5^th^ percentile ranges of ALT, AST and ALP than females. The mean values of the ALT, AST and ALP were also significantly different (p<0.05) between male and female. However, significant difference in gender was not observed in the mean values of LDH and amylase (p>0.05).

**Table 2:** Mean, median, 95% CI for mean and 2.5^th^-97.5^th^ percentile of clinical chemistry reference values in relation to sex of healthy adults in Gojjam, Zones, Amahra National Regional State, Northwest Ethiopia.

Parameters	Male				Female				Combined males and Females				
	Mean	Median	95% CI for mean	2.5^th^-97.5^th^ Percentile Range	Mean	Median	95% CI for mean	2.5^th^-97.5^th^ Percentile Range	P value	Mean	Median	95% CI for mean	2.5^th^-97.5^th^ Percentile Range
**ALT (U/L)**	20.0	18.5	18.9–21.2	6.0–44.6	16.1	15.0	15.1–17.0	3.0–30.0	0.000	18.0	17.0	17.3–18.8	6.0–43.0
**AST (U/L)**	24.1	23.5	23.1–25.1	10.5–39.0	19.4	19.0	18.6–20.2	6.0–32.1	0.000	21.7	21.0	21.1–22.4	9.0–38.0
**ALP (U/L)**	166.4	169.0	159.6–173.2	55.3–237.2	156.4	159.0	149.5–163.3	49.0–236.0	0.043	160.9	162.5	155.6–166.1	52.4–237.0
**Amylase (U/L)**	140.8	150.5	135.5–146.0	45.3–190.0	136.2	143.0	131.1–141.3	48.0–187.9	0.218	139.9	145.0	136–143.8	48.0–188.8
**LDH (U/L)**	331.0	356.0	319.6–343.2	146.1–402.0	315.3	349.0	301.1–329.6	137.7–405.1	0.085	323.6	354.0	314.4–329.8	145.0–403.0
**Creatinine (μmol/l)**	66.7	68.1	63.8–69.5	17.4–114.0	57.8	58.3	55.3–60.4	21.7–95.7	0.000	62.2	61.9	60.3–64.2	20.4–107.7
**Total Protein (g/l)**	70.2	70.2	68.9–71.5	53.0–86.7	70.1	71.0	68.8–71.5	53.2–86.0	0.607	69.1	69.0	68.1–70.2	53.0–86.1
**Total Bilirubin (μmol/l)**	18.5	16.2	17.3–19.7	4.7–37.6	17.7	15.3	16.4–18.9	3.6–37.6	0.105	17.6	15.1	16.7–18.6	4.4–37.6
**Direct Bilirubin (μmol/l)**	5.3	4.8	4.8–5.8	0.4–14.3	3.9	3.4	3.5–4.3	0.2–12.2	0.000	4.6	3.8	4.2–4.9	0.2–13.7

**Table 3:** Mean, median, 95% CI for mean and 2.5^th^-97.5^th^ percentile of clinical chemistry reference values in relation to age profile of healthy adults in Gojjam, Zones, Amahra National Regional State,Northwest Ethiopia.

Age (years)		ALT (U/L)	AST (U/L)	ALP (U/L)	Amylase (U/L)	LDH (U/L)	Creatinine (μmol/l)	Total Protein (g/l)	Total Bilirubin (μmol/l)	Direct Bilirubin (μmol/l)
**18–24**	**Mean**	16.8	21.5	160.2	141.6	319.6	61.9	68.1	17.3	4.1
	**Median**	16.0	21.0	163.0	147.0	355.0	61.9	67.2	15.4	3.4
	**95% CI for mean**	16.0–17.7	20.5–22.4	152.4–167.9	136.0–147.3	304.6–334.6	59.3–64.5	66.5–69.7	15.9–18.7	3.6–4.6
	**2.5**^**th**^**-97.5**^**th**^ **Percentile Range**	6.0–32.7	9.3–38.0	51.4–234.4	57.7–190.0	130.0–404.1	20.6–101.9	53.0–86.8	4.2–37.6	0.2–13.7
**25–34**	**Mean**	20.2	22.2	164.3	137.6	330.6	63.8	71.9	18.1	4.8
	**Median**	19.0	22.0	165.0	143.5	353.5	64.5	74.7	15.9	4.1
	**95% CI for mean**	18.5–22.0	20.8–23.6	154.9–173.7	129.4–145.7	312.8–348.4	60.1–67.5	69.8–73.9	16.4–19.8	4.2–5.5
	**2.5**^**th**^**-97.5**^**th**^ **Percentile Range**	5.0–45.0	7.0–39.0	48.8–238.2	20.8–187.1	158.5–402.5	9.7–111.4	52.9–86.7	3.7–36.1	0.2–13.7
**35–44**	**Mean**	19.0	21.6	158.0	138.8	317.1	59.0	68.2	18.8	4.5
	**Median**	17.0	20.5	157.0	144.0	348.0	58.3	67.7	14.7	3.9
	**95% CI for mean**	16.6–21.3	19.5–23.7	140.5–175.4	128.3–149.3	295.1–339.0	52.2–65.9	65.5–70.9	15.4–22.2	3.5–5.5
	**2.5**^**th**^**-97.5**^**th**^ **Percentile Range**	4.4–44.0	6.4–39.0	45.8–238.3	53.6–187.3	153.3–405.0	10.4–144.0	53.6–86.2	3.7–38.7	0.24–14.8
**45–54**	**Mean**	17.4	21.8	161.3	135.7	318.8	63.9	66.33	16.8	5.9
	**Median**	16.0	21.0	151.0	126.0	349.5	61.9	65.7	13.5	5.7
	**95% CI for mean**	14.2–20.7	19.3–24.3	144.1–178.6	121.7–149.7	287.4–350.1	56.2–71.5	62.7–70.0	12.8–20.9	4.1–7.6
	**2.5**^**th**^**-97.5**^**th**^ **Percentile Range**	3.0–35.0	9.0–38.0	93.0–232.0	57.0–181.0	132.0–403.0	23.0–114.9	53.0–82.2	7.7–37.6	0.2–16.4
**55–64**	**Mean**	14.2	21.4	144.7	136.1	345.2	62.7	66.6	15.1	6.5
	**Median**	14.0	21.0	142.0	141.0	364.0	59.2	63.8	14.19	5.6
	**95% CI for mean**	11.4–17.1	17.3–25.5	107.3–182.1	109.4–162.8	297.7–392.7	51.1–74.3	60.2–73.0	7.9–22.2	2.7–10.2
	**2.5**^**th**^**-97.5**^**th**^ **Percentile Range**	8.0–22.0	11.0–33.0	92.0–211.0	57.0–186.0	215.0–400.0	23.0–87.5	57.0–81.8	3.1–34.4	0.2–16.1
**≥ 65**	**Mean**	20.2	23.6	167.3	184.0	347.0	70.2	74.6	20.9	3.8
	**Median**	20.0	23.0	176.0	183.0	347.0	63.6	75.4	19.7	3.6
	**95% CI for mean**	15.3–25.1	20.4–26.8	41.73–292.9	177.4–190.6	136.0–358.0	33.0–107.0	70.0–79.3	5.1–47.0	2.6–4.9
	**2.5**^**th**^**-97.5**^**th**^ **Percentile Range**	15.0–25.0	21.0–28.0	113.0–213.0	71.0–187.0	325.0–369.0	43.3–114.9	72.5–76.0	11.1–32.0	2.2–6.8
**p-value**		**0.003**	**0.952**	**0.96**	**0.54**	**0.798**	**0.695**	**0.107**	**0.974**	**0.092**

**Table 4:** Mean, median, 95% CI for mean and 2.5^th^-97.5^th^ percentile of clinical chemistry reference values in relation to educational status of healthy adults in Gojjam, Zones, Amahra National Regional State, Northwest Ethiopia.

Parameters	Illiterate And Elementary Completed				High School Completed And Above				Both				
	Mean	Median	95% CI for mean	2.5^th^-97.5^th^ Percentile Range	Mean	Median	95% CI for mean	2.5^th^-97.5^th^ Percentile Range	P value	Mean	Median	95% CI for mean	2.5^th^-97.5^th^ Percentile Range
**ALT(U/L)**	17.7	16.0	16.4–18.9	5.0–44.0	18.3	18.0	17.3–19.2	6.0–42.5	**0.44**	18.0	17.0	17.3–18.8	6.0–43.0
**AST (U/L)**	21.7	21.0	20.6–22.8	8.0–39.0	21.8	21.0	20.9–22.6	9.6–38.0	**0.88**	21.7	21.0	21.1–22.4	9.0–38.0
**ALP (U/L)**	158.9	162.5	149.3–168.5	46.8–236.2	162.0	162.5	155.9–168.2	68.2–237.0	**0.347**	160.9	162.5	155.6–166.1	52.4–237.0
**Amylase(U/L)**	135.6	143.0	128.7–142.4	48.0–187.8	142.5	147.0	137.8–147.3	45.0–189.0	**0.119**	139.9	145.0	136.0–143.8	48.0–188.8
**LDH (U/L)**	316.3	346.5	303.0–329.5	147.6–402.1	330.1	356.0	317.3–343.0	141.8–405.4	**0.138**	323.6	354.0	314.4–329.8	145.0–403.0
**Creatinine (μmol/l)**	61.1	59.2	58.0–64.2	20.9–114.9	63.0	63.6	60.5–65.5	18.0–102.5	**0.34**	62.2	61.9	60.3–64.2	20.4–107.7
**Total Protein (g/l)**	69.1	69.0	67.5–70.7	53.5–85.9	69.2	68.8	67.8–70.6	53.0–86.8	**0.846**	69.1	69.0	68.1–70.2	53.0–86.1
**Total Bilirubin (μmol/l)**	17.9	14.7	16.2–19.5	4.3–37.6	17.5	15.4	16.3–18.7	4.2–37.1	**0.267**	17.6	15.1	16.7–18.6	4.4–37.6
**Direct Bilirubin (μmol/l)**	4.3	3.9	3.8–4.9	0.2–13.9	4.7	3.8	4.3–5.2	0.5–13.7	**0.418**	4.6	3.8	4.2–4.9	0.2–13.7

**Table 5:** Mean, median, 95% CI for mean and 2.5^th^-97.5^th^ percentile of clinical chemistry reference values in relation to monthly income of healthy adults in adults in Gojjam, Zones, Amahra National Regional State, Northwest Ethiopia.

Parameters	UP TO 1500ETB				>1500				Both				
	Mean	Median	95% CI for mean	2.5^th^-97.5^th^ Percentile Range	Mean	Median	95% CI for mean	2.5^th^-97.5^th^ Percentile Range	P value	Mean	Median	95% CI for mean	2.5^th^-97.5^th^ Percentile Range
**ALT(U/L)**	17.6	17.0	16.8–18.4	5.5–39.2	20.7	18.5	18.5–22.9	7.3–45.0	**0.004**	18.0	17.0	17.3–18.8	6.0–43.0
**AST (U/L)**	21.7	21.0	20.9–22.4	8.5–38.6	22.1	22.0	20.3–23.9	9.6–38.8	**0.65**	21.7	21.0	21.1–22.4	9.0–38.0
**ALP (U/L)**	160.6	163.5	154.7–166.4	49.0–236.0	162.4	155.5	150.7–174.2	77.2–240.0	**0.450**	160.9	162.5	155.6–166.1	52.4–237.0
**Amylase (U/L)**	138.9	144.0	134.5–143.2	47.1–189.0	145.3	149.5	135.7–154.9	36.7–189.0	**0.554**	139.9	145.0	136.0–143.8	48.0–188.8
**LDH (U/L)**	323.2	351.5	313.6–332.9	148.3–403.0	326.1	365.0	294.9–357.3	132.0–402.0	**0.842**	323.6	354.0	314.4–329.8	145.0–403.0
**Creatinine (μmol/l)**	61.4	61.0	59.3–63.5	20.8–107.0	67.3	70.3	62.3–72.3	8.2–112.7	**0.036**	62.2	61.9	60.3–64.2	20.4–107.7
**Total Protein (g/l)**	68.5	68.0	67.3–69.6	53.0–85.9	72.7	76.9	69.7–75.5	53.3–87.3	**0.001**	69.13	69.0	68.1–70.2	53.0–86.1
**Total Bilirubin (μmol/l)**	17.6	14.9	16.5–18.7	4.6–37.6	17.9	17.9	15.4–20.4	3.3–37.0	**0.816**	17.6	15.1	16.7–18.6	4.4–37.6
**Direct Bilirubin (μmol/l)**	4.5	3.8	4.2–4.9	0.2–13.7	4.7	3.3	3.7–5.7	0.6–15.7	**0.731**	4.6	3.8	4.2–4.9	0.2–13.7

**Table 6:** Mean, median, 95% CI for mean and 2.5^th^-97.5^th^ percentile of clinical chemistry reference values in relation to climate of healthy adults in Gojjam, Zones, Amahra National Regional State, Northwest Ethiopia.

Parameters	High land				Lowland				Both				
	Mean	Median	95% CI for mean	2.5^th^-97.5^th^ Percentile Range	Mean	Median	95% CI for mean	2.5^th^-97.5^th^ Percentile Range	P value	Mean	Median	95% CI for mean	2.5^th^-97.5^th^ Percentile Range
**ALT (U/L)**	19.1	17.0	17.9–20.4	6.3–44.7	17.3	17.0	16.4–18.2	5.0–38.0	**0.021**	18.0	17.0	17.3–18.8	6.0–43.0
**AST(U/L)**	21.2	21.0	20.2–22.2	9.0–38.7	22.1	21.0	21.0–23.0	8.0–38.3	**0.23**	21.7	21.0	21.1–22.4	9.0–38.0
**ALP (U/L)**	164.5	165.5	155.6–173.3	62.5–238.1	158.7	160.5	152.2–165.3	47.0–236.0	**0.029**	160.9	162.5	155.6–166.1	52.4–237.0
**Amylase (U/L)**	141.1	147.0	134.7–147.6	44.2–188.2	139.2	143.0	134.1–144.2	46.3–189.0	**0.109**	139.9	145.0	136–143.8	48.0–188.8
**LDH (U/L)**	328.5	349.0	308.5–348.6	154.0–403.0	322.6	356.0	312.3–333.0	141.0–403.0	**0.642**	323.6	354.0	314.4–329.8	145.0–403.0
**Creatinine (μmol/l)**	63.7	63.6	60.8–66.5	20.0–108.5	61.3	61.0	58.7–63.9	19.7–109.0	**0.25**	62.2	61.9	60.3–64.2	20.4–107.7
**Total Protein (g/l)**	72.84	76.9	71.0–74.7	53.0–87.0	66.9	66.5	65.7–68.1	53.1–84.8	**0.000**	69.13	69.0	68.1–70.2	53.0–86.1
**Total Bilirubin (μmol/l)**	18.4	16.4	16.9–20.0	3.5–35.9	17.2	14.9	15.9–18.4	4.4–37.6	**0.448**	17.6	15.1	16.7–18.6	4.4–37.6
**Direct Bilirubin (μmol/l)**	4.6	3.6	4.0–5.2	0.7–13.7	4.6	4.2	4.1–5.0	0.2–13.7	**0.489**	4.6	3.8	4.2–4.9	0.2–13.7

In the current study, there was no significant difference (p>0.05) in the mean values of AST, ALP, amylase and LDH across all age groups of participants except ALT (Table 3). As shown in Table 4, the high school completed and above groups have mean values of ALT 18.3U/L against illiterate and elementary completed groups of 17.7U/L, AST of 21.8U/L against illiterate and elementary completed groups of 21.7 U/L, ALP of 162.0U/L against illiterate and elementary completed groups of 158.9U/L, amylase of 142.5U/L against illiterate and elementary completed groups of 135.5U/L and LDH of 330.1U/L against illiterate and elementary completed groups of 316.3U/L. The current study indicated non-significant difference (p>0.05) in the mean values and reference intervals of ALT, AST, ALP, amylase and LDH between high school completed, and illiterate and elementary completed groups.

As shown in Table 5, the study showed a significant difference (p< 0.05) in the values of ALT between groups having monthly income up to 1500 ETB and greater than 1500 ETB. The values of AST, ALP, LDH and amylase did not show significant difference between the two groups (p>0.05). Relatively higher values were recorded in the groups with monthly income greater that 1500 ETB. The study also showed non-significant difference (p>0.05) in the values of AST, amylase and LDH between people living in lowland and highland residences. The values of the ALT and ALP showed significant difference (p<0.05) between subjects from lowland and highland residences, with values higher from those of highland (Table 6).

As shown in Table 2, participants had overall mean value of creatinine of 62.2μmol/l, total protein of 69.1g/l, total bilirubin of 17.6μmol/l and direct bilirubin of 4.6μmol/l. Males had reference interval of creatinine 17.4–114.0 μmol/l against females of 21.7–95.7 μmol/l, total protein of 53.0–86.7g/l against females of 53.2–86.0g/l, total bilirubin of 4.7–37.6 μmol/l against females of 3.6–37.6 μmol/l and direct bilirubin of 0.4–14.3 μmol/l against females of 0.2–12.2μmol/l. Males had significantly(p<0.05) higher 2.5^th^-97.5^th^ percentile ranges of creatinine and direct bilirubin. The difference in the 2.5^th^-97.5^th^ percentile ranges of total protein and total bilirubin were not statistically significant between males and females (p>0.05). The mean values of creatinine and direct bilirubin were significantly different between male and female (p<0.05), whereas significant gender difference was not observed in the mean values of total protein and total bilirubin (p˃0.05).

As shown in Table 3, significant difference in the mean values of all parameters except ALT were not observed across all age groups of participants (p>0.05). The high school completed and above groups have mean values of creatinine of 63.0 μmol/l against 61.1 μmol/l, total protein of 69.2 μmol/l against 69.1 μmol/l, total bilirubin of 17.5 μmol/l against 17.9 μmol/l and direct bilirubin of 4.7μmol/l against 4.3μmol/l of the illiterate and elementary completed groups. The current study indicated non-significant difference (p>0.05) in the values of creatinine, total protein, total bilirubin and direct bilirubin between high school completed and illiterate and elementary completed groups (Table 4). The result showed significant difference (p<0.05) in the values of total protein and creatinine, and non-significant difference (p>0.05) in values of total bilirubin and direct bilirubin between groups having monthly income up to 1500 ETB and greater than 1500 ETB. Relatively higher values were recorded in the groups with monthly income of greater that 1500 ETB (Table 5). The study also showed non-significant difference (p>0.05) in the values of creatinine, total bilirubin and direct bilirubin. Significantly (p<0.05) higher value of total protein was observed in people living in highland compared to those of lowland residences (Table 6).

## Discussion

Reference intervals for clinical chemistry parameters are vital for assessment of the health status of human population. They are used as a baseline data in clinical trials. Some of the parameters are also used as markers for diagnosis of diseases [6]. Reference values are also essential for assessment of disease, prognosis, drug response and recruitment of participants in studies like clinical trials [6]. However, there is scarcity of reference interval data in Ethiopia for the common clinical chemistry parameters for healthy adult population. Due to lack of locally derived reference values for the parameters, clinicians use reference values derived from western population. However, studies conducted in different African countries indicated differences between locally derived and western derived reference values [11, 12]. Other studies conducted in different African countries faced challenges in the exclusion of more participants in the clinical trial study by applying western derived reference values. For example, studies conducted in Uganda indicated 31% exclusion rate by using western derived intervals for recruitment of participants compared with 17% exclusion rate while using locally derived reference intervals [11]. Another study conducted in Kenya, faced 40% exclusion rate with application of western derived reference values compared with the locally derived reference values [12]. This is major indication in support of establishment of locally derived population based reference values for use in medical care and medical research.

Table 7 summarized the comparison in the clinical chemistry parameters reference values between different African countries and USA with the current study. The significant difference in gender (higher in male than females) in the reference values of ALT, AST and ALP in this study are consistent with reports from Botswana, Tanzania, Middle belt Ghana, Nigeria and Kenya [2, 6, 12–15]. The reference values of ALT and AST are lower in the current study as compared to the reports from Botswana, Tanzania, Middle belt Ghana, Nigeria, Kenya [2,6,12–16]. However, it is higher than those from USA [17]. The reference interval of ALP is higher in this study compared to those from Tanzania, Middle belt Ghana, South Ghana and USA [2, 13, 17, 18]. The reference interval of amylase in this study is higher than those from Botswana, Tanzania, Middle belt Ghana, Nigeria, Kenya and USA [2,6, 12–15,17]. The reference interval of LDH in the current study is higher than those from Kenya, Tanzania and USA [2, 12, 15–17], and lower than those from Middle belt Ghana [13].

**Table 7:** Comparison of reference range values of clinical chemistry parameters of the present study with other studies in African and western countries.

Parameters	Sex	Present study	Nigeria	Kenya	Tanzania	Southern Ghana	Middle Ghana	Botswana	USA
**ALT (U/L)**	Combined	18.0(6.0–43.0)	NA	9.6–52	8–48	NA	7.0–51	17.48(7.0–46)	0.0–35.0
	Male	20.0(6.0–44.6)	17.3–48.4	10.8–53.9	9–55	12–53	8.0–54	19.94(8–53)	0.0–35.0
	Female	16.1(3.0–30.0)	19–38	8.6–47	7–45	10–39	6.0–51	14.25(7–33)	0.0–35.0
**AST (U/L)**	Combined	21.7(9.0–38.0)	NA	13.8–42.3	14–48	NA	14–51	21.55(13.0–42.0)	0.0–35.0
	Male	24.1(10.5–39.0)	26.0–49.4	14.9–45.3	15–53	19–65	17–60	23.8(14–48)	0.0–35.0
	Female	19.4(6.0–32.1)	22–58.4	13.1–38.1	14–35	16–47	13–48	18.57(12–31)	0.0–35.0
**ALP (U/L)**	Combined	160.9(52.4–237.0)	NA	NA	46–158	NA	85–242	NA	30.0–120.0
	Male	166.4(55.3–237.2)	NA	NA	45–170	124–479	101–353	NA	30.0–120.0
	Female	156.4(49.0–236.0)	NA	NA	45–155	98–316	82–293	NA	30.0–120.0
**Amylase (U/L)**	Combined	139.9(48.0–188.8)	NA	38.3–163.0	43–164	NA	32–139	96.11(47.0–76.0)	60–180
	Male	140.8(45.3–190.0)	52.0–127.5	40–171.3	50–180	NA	NA	99.99(49–181)	NA
	Female	136.2(48.0–187.9)	55–122.4	36–147.8	42–160.4	NA	NA	90.99(46–162)	NA
**LDH (U/L)**	Combined	323.6(145.0–403.0)	NA	126.0–263.9	127–264	NA	223–681	NA	100–190
	Male	331.0(146.1–402.0)	NA	NA	125–268	NA	NA	NA	NA
	Female	315.3(137.7–405.1)	NA	NA	128–264	NA	NA	NA	NA
**Creatinine (μmol/l)**	Combined	62.2(20.4–107.7)	NA	NA	42–90.0	NA	NA	44.2–97.24	0.0–133
	Male	66.7(17.4–114.0)	85.8(76.3–111.1)	77 (62–106.0)	48.0–96.0	81.0–141.0	56–119	53.04–97.24	0.0–133
	Female	57.8(21.7–95.7)	79.3(63.0–117.8)	66(51–91)	40.0–81.0	70.0–121.0	47.0–110	44.2–70.7	0.0–133
**Total Protein (g/l)**	Combined	69.1(53.0–86.1)	NA		66.0–86.5	NA	51.0–87.0	NA	55.0–80.0
	Male	70.2(53.0–86.7)	NA	NA	67.2–85.2	NA	NA	NA	NA
	Female	70.1(53.2–86.0)	NA	NA	65.8–85.5	NA	NA	NA	NA
**Total Bilirubin (μmol/l)**	Combined	17.6(4.4–37.6)	NA	4.9–39.9	5.2–41.0	1.7–27.0	2.9–25.8	3.42–30.8	5.1–17.0
	Male	18.5(4.7–37.6)	6.8 (3.4–17.1)	12.2(5.6–42.9)	6.0–42.0	NA	NA	5.13–35.91	NA
	Female	17.7(3.6–37.6)	2.3 (0.3–10.6)	9.6(4.4–26.8)	4.5–31.3	NA	NA	3.42–22.23	NA
**Direct Bilirubin (μmol/l)**	Combined	4.6(0.2–13.7)	NA	1.1–8.8	0.7–8.2	3.4–10.3	0.8–4.0	1.7–6.84	1.7–5.1
	Male	5.3(0.4–14.3)	NA	NA	0.93–8.43	NA	NA	1.7–8.6	NA
	Female	3.9(0.2–12.2)	NA	NA	0.7–5.83	NA	NA	0.0–5.13	NA

The significant higher reference intervals of creatinine and direct bilurubin in males compared to females in the current study is consistent with reports from Botswana, Tanzania, Middle belt Ghana, Nigeria and Kenya [2, 6, 12–16]. The reference value of creatinine in the current study is higher than those from Botswana, Tanzania [2, 6]. But, it is lower than those from USA [17]. In this study, the reference value of total bilurubin is not significantly different between male and females. This is in agreement with reports from other African countries [12–16,18]. The reference interval for the total protein in this study is in agreement with reports from Botswana, Tanzania, Kenya, Middle belt Ghana and USA [2, 6, 12, 13, 15–17]. The total bilirubin reference interval of the current study is higher than those from Botswana, Middle belt Ghana, South Ghana and USA [6, 13, 17, 18]. However, it is parallel with those from Tanzania and Kenya [2,12,15,16]. The direct bilirubin reference interval of the current study is higher than those from Botswana, Tanzania, South Ghana, Middle belt Ghana, Kenya and USA [2,6,12,13,15,17,18].

Significant difference in some of the parameters between groups with monthly income up to 1500 ETB and greater than 1500 ETB might be because of the life style variation and nutritional difference between the two groups. Similarly, differences were recorded between people living in low and high land residences. This might be due to geographical, environmental and life style variation between the two groups.

## Conclusion

In the current study some of the common clinical chemistry parameters reference interval of healthy adults in Gojjam zones showed some variation from the references values generated from western population. The overall reference intervals of ALT, AST, ALP, amylase, LDH, total and direct bilirubin for adult population were higher than those adopted from the developed nations. There was also variation of reference value based on climate, gender, monthly income and geographical locations. Therefore, further study is required to establish reference intervals for Ethiopian population.

## Abbreviations

ALP: alkaline phosphatise
ALT: alanine amino transferase
ANOVA: Analysis of Variance
ARPHI: Amhara region public health
AST: aspartate amino transferase
BMI: body mass index
CV: Coefficient of variation
EPHI: Ethiopia public health institute
ETB: Ethiopian Birr
g/l: gram per litter
HBsAg: Hepatitis B surface antigen
HBV: hepatitis B virus
HCG: Human Chorionic Gonadotropin
HCV: hepatitis C virus
HIV: human immune deficiency virus
LDH: Lactate dehydrogenase
NA: not available
NCCLS: National Committee for Clinical Laboratory Standards
NORIP: Nordic Reference Interval Project
QC: quality control
SD: standard deviation
Sq.km: square kilo meter
U/L: units per litter
WHO: world health organization
μ: mean of population
μmol/l: micro mole per litter
